# Rewriting the brain: m6A RNA methylation as an emerging epitranscriptomic regulator in major depressive disorder

**DOI:** 10.3389/fnmol.2026.1922816

**Published:** 2026-08-28

**Authors:** Anuj K. Verma, Hiroaki Mori, Bhaskar Roy, Yogesh Dwivedi

**Affiliations:** Department of Psychiatry and Behavioral Neurobiology, Heersink School of Medicine, University of Alabama at Birmingham, Birmingham, AL, United States

**Keywords:** depression, human brain, epitranscriptomics, m6A RNA methylation, plasticity, RNA modification, stress

## Abstract

Activity-dependent gene regulation is fundamental to synaptic plasticity, and its disruption is increasingly recognized as a feature of major depressive disorder (MDD). Environmentally driven changes in gene expression can alter neural plasticity in corticolimbic brain regions, yet the post-transcriptional mechanisms linking environmental stress to maladaptive neuronal function remain incompletely understood. RNA epitranscriptomic regulation has recently emerged as an important layer of gene control, with N6-methyladenosine (m6A) representing the most abundant and dynamically reversible internal modification of mammalian mRNA. Widely present in the adult brain, m6A regulates RNA splicing, export, stability, localization, and translation, thereby shaping transcript fate and protein output. Although m6A RNA methylation has been studied extensively in other biological contexts, its contribution to MDD pathophysiology is only beginning to be defined. Current human evidence is primarily correlative, derived largely from bulk-tissue postmortem datasets, and should be considered hypothesis-generating until replicated in independent cohorts and validated with cell-type-resolved and mechanistic approaches. Emerging clinical and preclinical studies suggest that dysregulated m6A signaling may influence neurodevelopmental, neurocognitive, and stress-responsive pathways relevant to depression. By integrating environmental signals with transcriptomic regulation, m6A modification may represent a plausible epitranscriptomic mechanism governing synaptic plasticity in the depressed brain. In this review, we summarize the dynamic regulation of m6A RNA methylation in the brain, discuss its neurobiological functions, and critically evaluate its potential role in stress-related pathology and MDD.

## 1 Introduction

### 1.1 Burden and clinical complexity of major depression

Major depressive disorder is a highly prevalent and clinically heterogeneous psychiatric condition associated with sustained allostatic burden within corticolimbic networks of the human brain (McEwen and Gianaros, 2010; Finlay et al., 2025). Reduced adaptive capacity in response to stress, shaped by socioeconomic adversity and genetic vulnerability, may drive maladaptive neural responses across corticolimbic and interconnected brain regions (McEwen and Gianaros, 2010; McEwen and Morrison, 2013; Finlay et al., 2025; Lei et al., 2025). These perturbations are associated with progressive structural and functional remodeling of stress- and emotion-regulatory circuits (McEwen and Morrison, 2013; McEwen et al., 2016; Finlay et al., 2025; Lei et al., 2025). Collectively, such changes may dismantle the structural and functional plasticity required for neuronal resilience and adaptive behavioral regulation (McEwen et al., 2016; Finlay et al., 2025).

### 1.2 Synaptic plasticity and gene regulation

Neural plasticity provides a conceptual framework for understanding how neural networks integrate, adapt to, and encode signals arising from both external and internal environments; disruption of this capacity may compromise information transfer and coordination across neural networks (Citri and Malenka, 2008; McEwen et al., 2016). Deficits in neural plasticity are therefore increasingly viewed as core biological features of mood dysregulation, particularly in major depressive disorder (MDD) (Citri and Malenka, 2008; Duman and Aghajanian, 2012; McEwen et al., 2016; Price and Duman, 2020). At the cellular level, neural plasticity is mediated largely by activity-dependent structural and functional remodeling within the synaptic milieu, collectively referred to as synaptic plasticity (Citri and Malenka, 2008; McEwen et al., 2016). Convergent evidence further indicates that transcriptome-wide alterations in gene expression are central to aberrant synaptic plasticity in the depressed brain (Duman and Aghajanian, 2012; Duric et al., 2013; Price and Duman, 2020; Yoshino et al., 2021). Although inherited genetic vulnerability may contribute to these expression patterns, the substantial discordance observed in twin studies underscores the importance of non-genetic regulatory mechanisms in shaping MDD pathophysiology (McGuffin et al., 1996).

### 1.3 Beyond genetics: emergence of RNA epitranscriptomics

Beyond canonical epigenetic mechanisms, emerging evidence highlights methylation-based covalent RNA modifications as a dynamic post-transcriptional regulatory layer with broad consequences for gene expression (Roundtree et al., 2017; Jiang et al., 2021). Such RNA-level regulatory processes may be particularly relevant to the molecular dysregulation underlying reduced synaptic plasticity in the MDD brain (Roy et al., 2022; Roy and Dwivedi, 2025). Because these modifications involve reversible enzymatic remodeling of coding and non-coding RNA molecules, with downstream effects on RNA fate and protein output, they are increasingly recognized as key epitranscriptomic mechanisms through which cells maintain functional adaptability (Roundtree et al., 2017; Jiang et al., 2021). Disruption of this finely balanced regulatory system may therefore contribute to pathogenic processes, as supported by accumulating evidence from both clinical and preclinical studies (Jiang et al., 2021; Xia et al., 2024; Wang et al., 2025).

### 1.4 Scope and aim

This review provides a critical synthesis of emerging evidence positioning m6A-based epitranscriptomic modification as a pivotal regulatory mechanism across normal and pathological brain states. By framing m6A-mediated RNA regulation as a dynamic molecular interface linking gene expression programs, synaptic plasticity, and neural circuit adaptation, the review delineates its potential significance in neuropsychiatric disorders, with particular emphasis on stress-related pathology and MDD (Jiang et al., 2021; Roy et al., 2022; Xia et al., 2024; Roy and Dwivedi, 2025; Wang et al., 2025).

## 2 Generalized overview of m6A-based RNA methylation machinery and mode of action

### 2.1 Chemical nature and distribution of m6A modification

With recent technological advancements, whole-transcriptome-based *de novo* sequencing has revealed information on more than 150 types of RNA modifications at the post-transcriptional level (Boccaletto et al., 2022; Zhang et al., 2022). One of the most well-characterized RNA base modifications is RNA editing, which involves the enzymatic deamination of adenosine to inosine (Slotkin and Nishikura, 2013). Adenosine residues can also be methylated to m6A, which is quite analogous to the DNA-based cytosine methylation. Transcriptome-wide m6A mapping technologies have shown that m6A residues are present in at least 8,000 transcripts, often near the stop codons, in the 3’UTR or in the 5’UTR of transcripts (Dominissini et al., 2012; Meyer et al., 2012). This, along with the recent mapping of 5-methylcytosine sites in mammalian mRNA, points to a diverse “epitranscriptomic” landscape that influences mRNA fate and function in cells (Squires et al., 2012; Hussain et al., 2013). However, the methylation-based RNA modification, which appears to occur at the 6th nitrogen position of adenosine (N6-methyladenosine), has been reported to produce overall epitranscriptomic changes by either modulating transcript turnover rates or generating splice variants (Wang X. et al., 2014; Meyer and Jaffrey, 2017).

### 2.2 The core mechanism

**Figure 1:**
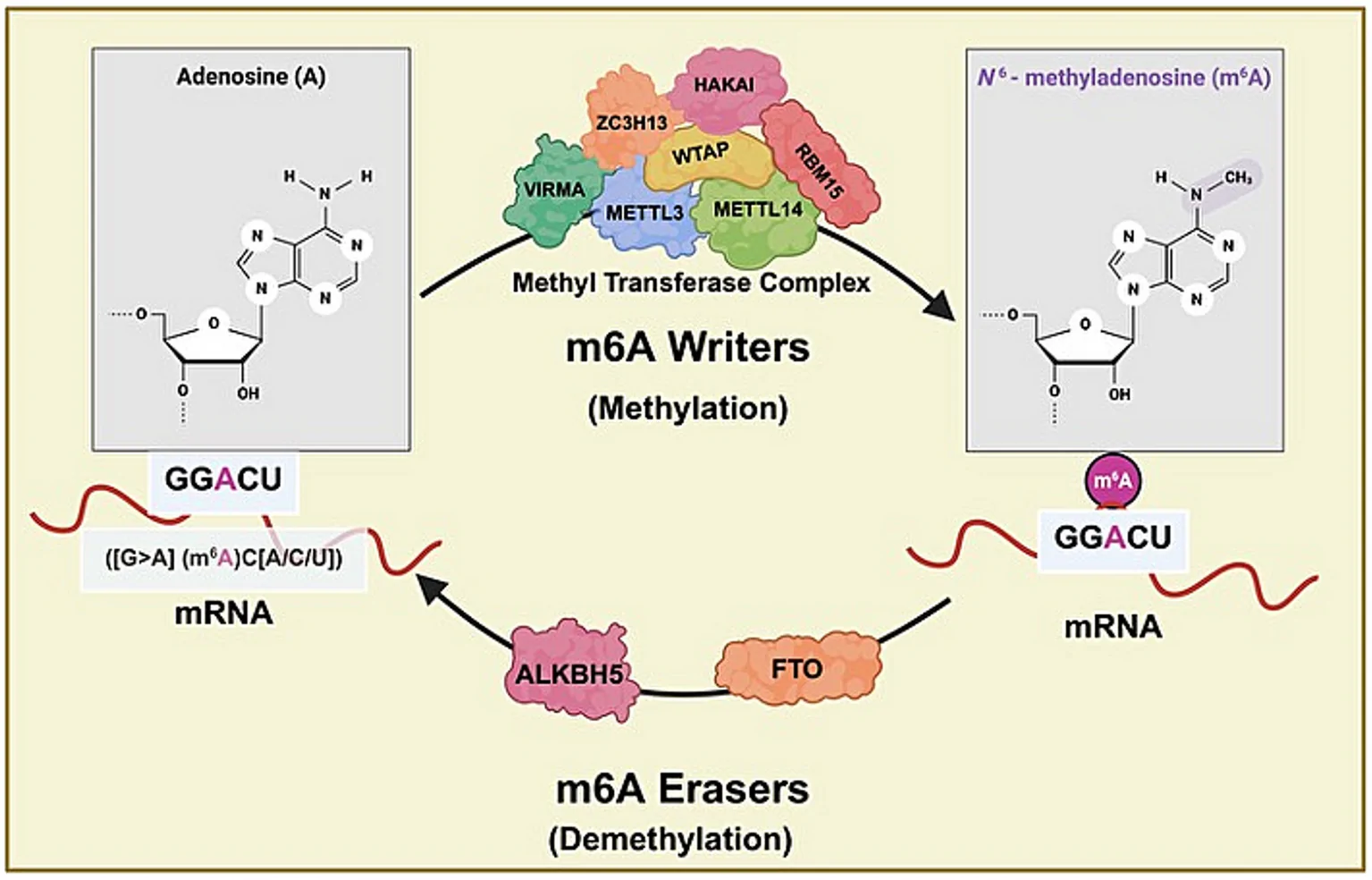
Methylation machinery. A methyl group is added by m6A writers (METTL3 and METTL14) to the N6-position of adenosine within the consensus sequence ([G > A](m6A)C[A/C/U]) of an mRNA transcript. The m6A erasers are FTO and ALKBH5. ALKBH5 and FTO are m6A demethylases that remove the methyl group from N6-adenosine from the target mRNA. Using by BioRender.

The reversible nature of this m6A methylation is dynamically modulated by a complex network of proteins called “writers” or methylases [METTL3 (methyltransferase-like 3) and METTL14 (methyltransferase-like 14)], “erasers” or demethylases [FTO (fat mass and obesity-associated protein) and ALKBH5 (alkB homolog 5)], and “readers” [YTHDF1, YTHDF2, YTHDF3 (YTH N^6^-methyladenosine RNA-binding proteins F1-F3), as well as YTHDC1 and YTHDC2 (YTH domain-containing proteins C1 and C2)] (Meyer and Jaffrey, 2017; Roundtree et al., 2017; Jiang et al., 2021). m6A methylation is installed by the methyltransferase complex (MTC), comprising the core catalytic components METTL3, METTL14, and WTAP (Wilms tumor 1-associating protein), together with regulatory subunits including VIRMA/KIAA1429 (Vir-like m6A methyltransferase associated), ZC3H13 (zinc finger CCCH-type containing 13), HAKAI/CBLL1 (Cbl proto-oncogene-like 1), and RBM15 (RNA-binding motif protein 15) (Meyer and Jaffrey, 2017; Roundtree et al., 2017; Jiang et al., 2021). Functionally, METTL3 provides the principal catalytic activity, whereas METTL14 has a non-redundant structural and RNA-binding role that stabilizes METTL3, enhances substrate recognition, and helps position RNA within the writer complex (Śledź and Jinek, 2016; Yoon et al., 2017). WTAP serves as a scaffold that supports nuclear localization and efficient activity of the complex, while accessory factors help confer target specificity and regional methylation preferences (Meyer and Jaffrey, 2017; Roundtree et al., 2017; Jiang et al., 2021). WTAP, together with METTL3, also contributes to the regulation of gene expression and alternative splicing. A concise overview of the major m6A writers, erasers, and readers, together with their principal functions in RNA metabolism, is provided in Table 1 and Figure 1.

**Table 1:** Functional roles of m6A regulators in RNA metabolism.

Type	m6A regulator	Function
m6A writers	METTL3	Catalyze m6A modification
	METTL14	Non-redundant structural and RNA-binding component that stabilizes METTL3, enhances substrate recognition, and supports methyltransferase-complex activity
	WTAP	Act as a scaffold protein and is important for nuclear localization of the complex
	VIRMA	Helps in recruiting core methylation complex to guide methylation of a particular region
	ZC3H13	Acts as a structural anchor for WTAP to the mRNA-binding factor in the nucleus
	HAKAI	Required for stabilization of core components of m6A methylation machinery
	RBM15	Recruit the methylation complex to a specific site of RNA
m6A erasers	FTO	Catalyze m6A demethylation
	ALKBH5	Remove m6A methylation
Nuclear m6A readers	YTHDC1	Regulates the mRNA splicing, export and nuclear retention
	HNRNPs	Recognizes m6A sites on nascent transcript to regulate alternate splicing
Cytosolic m6A readers	YTHDF1	Primarily enhances translation
	YTHDF2	Promotes the mRNA degradation
	YTHDF3	Work with both YTHDF1 and YTHDF2 to coordinate the translation process and mRNA decay
	YTHDC2	Enhances translation of target RNA
	IGF2BPs	Enhances translation and mRNA stability

Demethylases, or erasers, such as FTO and ALKBH5 remove methyl marks from RNA. Both proteins belong to the alpha-ketoglutarate-dependent dioxygenase family (Jia et al., 2011; Zheng et al., 2013) and are broadly expressed, although their activity varies across tissues. FTO, which is highly expressed in the brain, was initially described as an m6A demethylase, but subsequent work indicates that its substrate specificity can include cap-adjacent N6, 2′-O-dimethyladenosine (m6Am) as well as internal m6A, depending on transcript context, cellular compartment, and assay conditions (Mauer et al., 2017; Wei et al., 2018). Therefore, reduced FTO expression should not be interpreted automatically as global m6A accumulation without direct modification mapping. Li et al. further reported that FTO depletion increases methylation on mRNAs involved in ribosome biogenesis (Li et al., 2025). ALKBH5, the second identified m6A demethylase, regulates mRNA export, RNA metabolism, and gene expression by reducing m6A marks (Jiang et al., 2021; Ngo et al., 2025).

Reader proteins recognize and bind to the m6A-modified RNA and regulate gene expression through mRNA stability, mRNA splicing, mRNA export, miRNA biogenesis, and translation efficiency (Patil et al., 2018; Han et al., 2021). Different readers exert distinct functions depending on their cellular localization and the position of m6A marks. Nuclear m6A readers include YTHDC1 and heterogeneous nuclear ribonucleoproteins (HNRNPs), such as HNRNPA2B1 and HNRNPC11. YTHDC1 regulates mRNA splicing, export, and nuclear retention, whereas HNRNPs recognize m6A sites on nascent transcripts to modulate alternative splicing. Cytoplasmic m6A readers include YTHDF1, YTHDF2, YTHDF3, YTHDC2, and IGF2BP1/2/3 (insulin-like growth factor-2 mRNA-binding proteins). YTHDF1 primarily enhances translation, YTHDF2 promotes mRNA decay, and YTHDF3 functions as a coordinator that cooperates with YTHDF1 and YTHDF2 to regulate translation and mRNA turnover. IGF2BPs enhance mRNA stability by binding m6A motifs in target transcripts.

Importantly, the functional consequences of m6A are reader-dependent rather than uniform. The same methylated transcript may exhibit altered stability, translation, localization, or decay depending on which reader proteins are expressed, where the m6A site occurs, and the cellular state (Meyer and Jaffrey, 2017; Patil et al., 2018; Zaccara et al., 2019). This distinction is especially relevant for stress- and plasticity-related transcripts such as *Nr3c1*, *Creb1*, and *Ntrk2*, for which m6A enrichment may plausibly influence glucocorticoid signaling, CREB-dependent transcriptional programs, or BDNF/TrkB-associated plasticity through changes in RNA fate rather than through a single deterministic mechanism.

YTHDF1 is a cytoplasmic m6A reader protein that selectively recognizes m6A sites through its carboxy-terminal YTH domain. Once bound to the target RNA, YTHDF1 recruits translation initiation factors like eIF3a and eIF3b via its amino-terminal region. These initiation factors play a crucial role in assembling the 43S pre-initiation complex and recruiting the 40S ribosomal subunit to mRNA, thereby initiating translation. Consistent with this role, YTHDF1 knockdown reduces ribosome occupancy on target mRNAs, including at translation initiation sites (Chen et al., 2021).

YTHDF2 is another m6A methylation reader protein, which is responsible for RNA decay. Under stress conditions, m6A-modified RNA–YTHDF2 complexes can partition into intracellular compartments, including P bodies, stress granules, and neuronal RNA granules, where RNA degradation is facilitated. YTHDF2 can also recruit the CCR4-NOT deadenylase complex to m6A-modified RNA, accelerating poly(A) shortening independently of P-body components. This process involves recruitment of the CCR4-NOT complex through the N-terminal region of YTHDF2 and the SH domain of CNOT1, the scaffold subunit of the CCR4-NOT complex (Lee et al., 2020).

**Figure 2:**
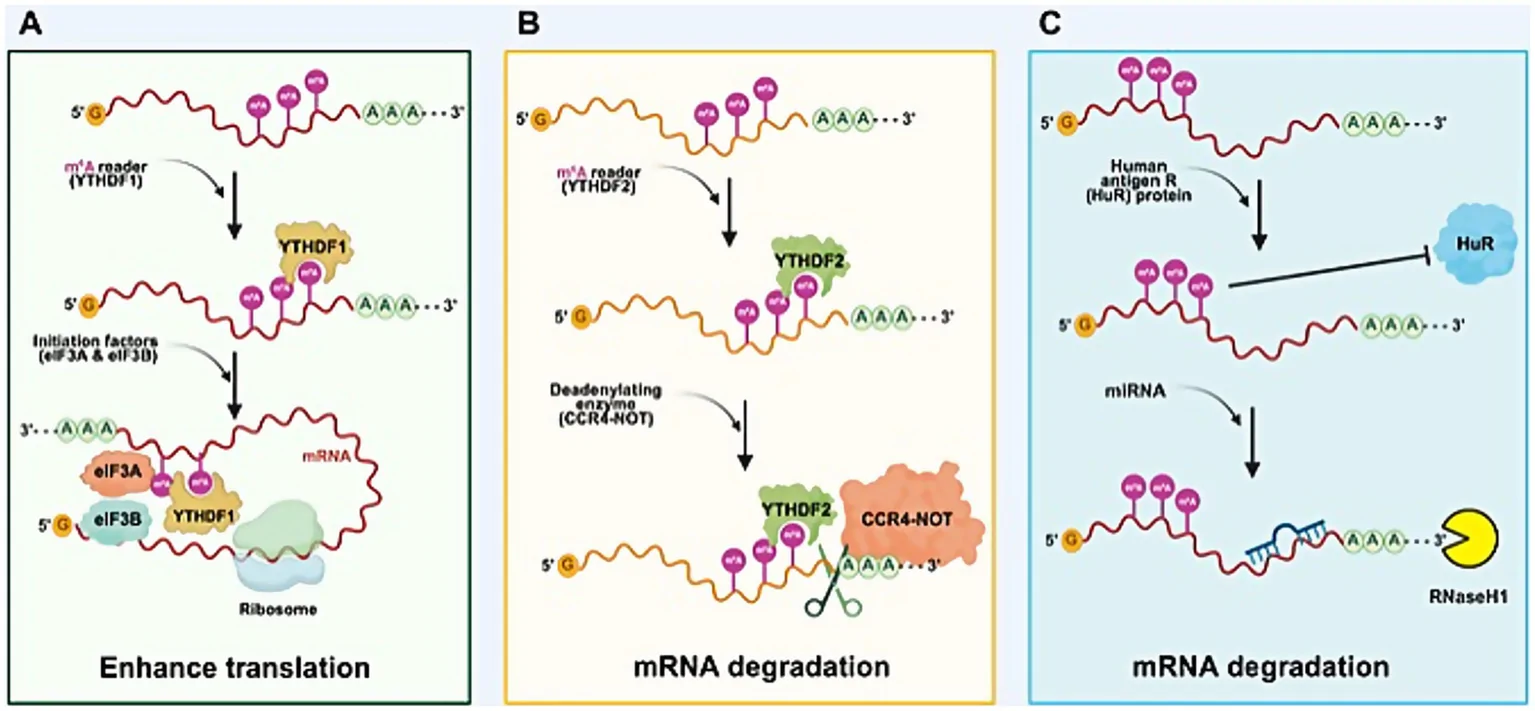
Mode of action of m^6^A methylation**. (A)** YTHDF1 is involved in mRNA translation. YTHDF1 promotes the translation of m^6^A-modified mRNA by interacting with the eukaryotic translation initiation factors eIF3A and eIF3B. These initiation factors recruit the ribosomal complex and initiate translation. **(B)** YTHDF2 is a major m^6^A reader involved in the degradation of m^6^A-modified RNAs. YTHDF2 directly recruits the CCR4-NOT deadenylase complex to m^6^A-modified mRNAs, leading to deadenylation and degradation of mRNAs. **(C)** HuR binding blocks mRNA decay. m^6^A methylation blocks HuR binding when the m^6^A site is sufficiently far from the HuR binding site. This leads to miRNA-mediated decay of m^6^A-methylated mRNA BioRender.

Additional evidence indicates that m6A RNA methylation exerts selective effects on RNA metabolism. For example, methylation within the 3’UTR can prevent binding of the RNA-stabilizing protein HuR to methylated transcripts (Kundu et al., 2012). This selective exclusion of HuR binding from its mRNA makes the mRNA molecule more prone to microRNA-mediated degradation (Kundu et al., 2012; Wang Y. et al., 2014). Due to this dynamic mode of regulation, cellular physiology makes this RNA modification process a potential target and is invariably driven by extrinsic environmental factors as well as intrinsic physiological and cellular milieu (Wang X. et al., 2014; Meyer and Jaffrey, 2017). But most interestingly, because this type of chemical alteration in the mRNA molecule is an immediate contributor to protein synthesis pathways, it can induce dynamic changes in cellular activity (Wang X. et al., 2014; Meyer and Jaffrey, 2017). This could be considered a rapid mode of response to environmental stimuli and can produce far more diverse effects than other epigenetic mechanisms (Wang X. et al., 2014; Meyer and Jaffrey, 2017). Various modes of action of m6A are provided in Figure 2.

## 3 Neurobiological outlook of m6A-based RNA epitranscriptomic modification

Over the past few years, a considerable number of reports on m6A methylation have shaped our understanding of this unique epitranscriptomic modification in biological processes ranging from development to disease (Meyer and Jaffrey, 2017; Roundtree et al., 2017). However, its neurobiological roles remain less well characterized than its functions in other biological contexts. Several studies have suggested that this dynamic RNA modification contributes to CNS function, including neurogenesis, memory formation, dopaminergic signaling, and stress-related behavioral regulation (Hess et al., 2013; Yoon et al., 2017; Engel et al., 2018). Nevertheless, the broader functional significance of RNA-level epitranscriptomic modification remains less understood, particularly in neuropsychiatric disorders.

An early study using dopaminergic neuron-specific Fto knockout mice demonstrated impaired D2 receptor-mediated dopaminergic signaling. Mechanistically, loss of Fto disrupted RNA demethylation and attenuated G protein-coupled receptor activation in conditional knockout mice. This impairment was linked to dysfunction of D2-like receptor signaling within midbrain dopaminergic circuitry, with potential consequences for complex behavioral regulation (Hess et al., 2013). This was the first study to identify the role of m6A RNA methylation and its dysfunctionality in behavioral impairment in a genetic animal model.

Earlier, a genetic association study implicated FTO in MDD, although it primarily examined FTO variants associated with altered body mass index (BMI) in a large cohort of MDD cases and controls (Rivera et al., 2012). Replication in an independent MDD cohort supported the authors’ hypothesis of shared genetic liability between obesity and depression, suggesting a possible mechanistic role for FTO in both conditions. This evidence should be interpreted as genetic association rather than direct evidence of altered FTO enzymatic activity or transcript-specific m6A/m6Am remodeling in MDD. Beyond FTO-BMI-related evidence, candidate-gene studies have more directly examined m6A-related genes in MDD. In a Chinese Han population, rs12936694 in ALKBH5, an m6A demethylase, was associated with MDD (Du et al., 2015). More recently, ELAVL1 and YTHDC2 were associated with MDD, and ELAVL1 was additionally associated with anxiety symptoms in MDD (Li et al., 2024). Collectively, these studies suggest that m6A-related regulatory pathways may contribute to MDD vulnerability, although the evidence remains indirect, relying on genetic associations rather than direct measurements of brain m6A or m6Am modification.

Over the past 10 years, the important role of m6A methylation in neurobiological contexts has gradually been clarified. The role of m6A methylation in cortical neurogenesis has been demonstrated using the Mettl14 knockout mouse model (Yoon et al., 2017). Additionally, a transcriptome-wide m6A methylation profile in the mouse brain has revealed heterogeneity in methylation patterns across brain regions, as well as differential preferences for methylation sites, resulting in variability in biological functions (Chang et al., 2017). Altered expression of the demethylase Fto has also been linked to defects in postnatal neurogenesis (Li et al., 2017). Furthermore, contextual fear conditioning has been shown to reduce Fto expression in the nuclei, dendrites, and areas near dendritic spines of mouse dorsal hippocampal CA1 neurons. Together, these implicate the role of Fto in memory formation (Walters et al., 2017). In an Fto-knockdown mouse model, the medial prefrontal cortex was found to be responsive, enhancing consolidated fear memory by increasing m6A methylation at specific gene transcripts (Engel et al., 2018). Fto inactivation also impaired dopamine receptor type 2 (D2R) signaling, while Fto depletion in the midbrain and striatum increased adenosine methylation in mRNAs involved in learning, reward behavior, motor function, and feeding (Hess et al., 2013).

Recently, several studies using animal models have provided important support for the involvement of m6A RNA modification in the biology of depression. One study demonstrated that hippocampal FTO mediates depression-like behaviors through RNA demethylase activity (Liu et al., 2021). In this study, knocking down or knocking out FTO in the hippocampus induced depression-like behaviors, whereas FTO overexpression exerted antidepressant-like effects. The study further suggested that Adrb2 mRNA is a downstream target regulated by FTO-dependent m6A demethylation. In addition, a bioinformatic analysis using rat models of depression reported that m6A regulator-mediated RNA methylation modification patterns were associated with the pathogenesis and immune microenvironment of depression (Wang et al., 2022). Together, these findings suggest a causal relationship between altered FTO-dependent m6A demethylation and depression-like behavioral phenotypes *in vivo*.

**Figure 3:**
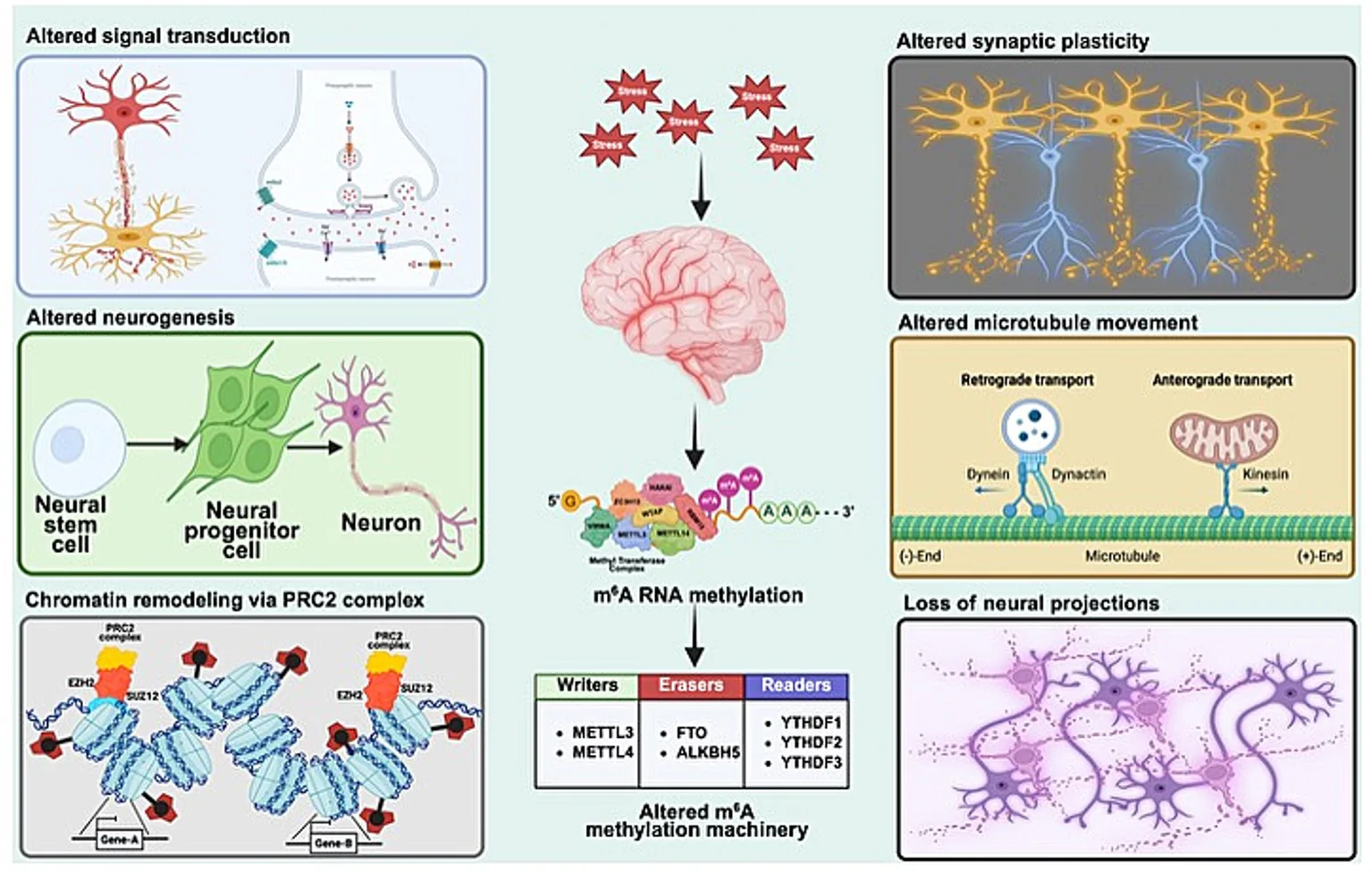
m6A methylation machinery and its neuronal functions. Schematic overview illustrating how N6-methyladenosine (m6A) RNA methylation, controlled by writers (METTL3, METTL14, WTAP and associated regulatory factors), erasers (FTO, ALKBH5), and readers (YTHDF1, YTHDF2, YTHDF3 and related m6A-binding proteins), regulates key functions of the Central Nervous System (CNS). Dysregulation of this machinery converges on neuronal and glial processes, including altered signal transduction, neurogenesis, chromatin remodeling, synaptic plasticity, microtubule-based axonal transport, and loss of neuronal projections. METTL4 is not considered a canonical m6A writer in this review and should not be interpreted as part of the METTL3/METTL14 writer complex unless explicitly specified in the figure artwork BioRender.

The monoamine hypothesis of depression, particularly the serotonin-CREB-BDNF pathway, has received extensive attention in relation to MDD pathophysiology. However, the post-transcriptional regulation of candidate genes within serotonergic and plasticity-related pathways remains poorly understood. A genome-wide association study identified a strong correlation between the incidence of MDD and 10 single-nucleotide polymorphisms (SNPs) in the FTO gene, an RNA demethylase reported to be expressed in the CNS at higher levels in normal individuals (Rivera et al., 2012). Although the functional effects of these FTO variants on depression-related molecular pathways remain unclear, they raise the possibility that altered RNA demethylation may distinguish disease from non-disease states. Such changes could affect the stability or translational fate of candidate transcripts such as BDNF and CREB, potentially through P-body-mediated decay or miRNA-induced silencing complex-dependent degradation. This hypothesis is biologically plausible and is supported by our learned helplessness rat study, in which stress was associated with downregulation of Fto, upregulation of Mettl3, and m6A enrichment in plasticity-related transcripts such as Nr3c1, Creb1, and Ntrk2 in the hippocampus (Roy et al., 2022). However, these findings derive from an animal stress model and should be interpreted as preliminary until validated across additional models, sexes, brain regions, developmental windows, and independent laboratories. The data link m6A dysregulation to glucocorticoid signaling, CREB-dependent transcriptional regulation, and BDNF/TrkB-related plasticity pathways, all of which are highly relevant to depression biology; nevertheless, direct causal evidence that m6A remodeling of BDNF/TrkB signaling drives human MDD remains limited. In another study, hypericin ameliorated depression-like behaviors in a chronic unpredictable mild stress model and was reported to regulate METTL3 and WTAP expression, m6A modification, and neurotrophin signaling pathways (Lei et al., 2023). Together, these data suggest that m6A-related post-transcriptional regulation may influence plasticity-related molecular cascades that overlap with classical CREB-BDNF-based models of depression. Key human genetic, postmortem, clinical, and preclinical studies implicating m6A RNA methylation in depression and depression related phenotypes are summarized in Table 2 and Figure 3.

**Table 2:** Human postmortem brain, clinical and preclinical studies associated with m6A RNA methylation changes.

Species	Category	m6A methylation/m6Aregulator	Research finding	Reference
Human	Candidate-gene association study	FTO polymorphisms	A history of depression moderates the effect of the FTO gene on BMI	Rivera et al. (2012)
Human	Candidate-gene association study	ALKBH5 rs12936694 polymorphism	ALKBH5 rs12936694 was associated with MDD in a Chinese Han population.	Du et al. (2015)
Mouse/Human	Acute stress model mouse and Human MDD blood m6A-seq study	Transcriptome-wide m6A methylation	Linked m6A/m RNA methylation to stress-response regulation and reported altered m6A/m dynamics in MDD-relevant human material.	Engel et al. (2018)
Mouse/Human	Animal model of depression/human postmortem brain and blood	m6A regulators, including FTO	Hippocampal FTO regulated depression-like behaviors via RNA demethylase activity; FTO was also reported to be reduced in MDD hippocampal samples.	Liu et al. (2021)
Rat	Animal model of depression	m6A regulators, including Fto and Mettl3	Learned helplessness rats showed Fto downregulation, Mettl3 upregulation, and m6A enrichment in plasticity-related transcripts such as Nr3c1, Creb1, and Ntrk2.	Roy et al. (2022)
Rat	In silico analysis of animal model of depression	m6A regulators	Identified m6A regulator-mediated modification patterns associated with depression pathogenesis and immune microenvironment features.	Wang et al. (2022)
Mouse	Animal model of depression	METTL3, WTAP, and m6A methylation	Hypericin ameliorated depression-like behaviors in a chronic unpredictable mild stress model and regulated METTL3/WTAP, m6A modification, and neurotrophin signaling.	Lei et al. (2023)
Human	Candidate-gene association study	m6A-related genes polymorphisms including ELAVL1 and YTHDC2	ELAVL1 and YTHDC2 were associated with MDD; ELAVL1 was also associated with anxiety symptoms in MDD.	Li et al. (2024)
Human	In silico analysis	m6A/m1A/m5C-associated methylation regulators	Integrated m6A/m1A/m5C regulators and identified RNA-methylation-related gene signatures and immune-profile alterations in MDD.	Ren et al. (2024)
Human	In silico analysis	m6A regulators	m6A regulators may contribute to neuropsychiatric disease susceptibility through effects on gene expression and protein regulation.	Liufu et al. (2024)
Human	Human postmortem brain m6A-seq study	Transcriptome-wide m6A methylation	Direct m6A-seq analysis identified sex-dependent m6A dysregulation in the MDD vmPFC, with altered cellular-function-related pathways.	Mitsuhashi et al. (2024)
Human	Human postmortem brain m6A microarray study	Transcriptome-wide m6A methylation	Direct m6A epitranscriptome profiling identified altered m6A methylation, reduced FTO, increased METTL3, and suicide-risk-related m6A signatures in MDD.	Roy and Dwivedi (2025)
Human/Review	Review of stress-responsive neurodegeneration signaling	c-Jun/AP-1 and JNK-related signaling	Highlighted c-Jun as a context-dependent regulator of oxidative stress, inflammation, apoptosis, axonal remodeling, and neuronal adaptation, providing broader stress-signaling context relevant to neuropsychiatric vulnerability.	Khan et al. (2026)

MDD, major depressive disorder; BMI, body mass index; vmPFC, Ventromedial prefrontal cortex.

Recent in silico studies suggest that RNA methylation-related regulators may contribute to MDD through immune and multi-omics regulatory pathways. One study integrating m6A/m1A/m5C-associated methylation regulators identified RNA-modification-related gene signatures and immune-profile alterations in MDD, suggesting that RNA methylation may be linked to immune-cell infiltration and inflammatory pathways in depression (Ren et al., 2024). In addition, a multi-omics summary-data integration study across psychiatric and neurodegenerative disorders, including MDD, suggested that m6A regulators may influence disease susceptibility through effects on gene expression and protein regulation, providing broader genetic and functional support for m6A involvement in neuropsychiatric diseases (Liufu et al., 2024). Recent literature further broadens this context by emphasizing that RNA-regulatory and stress-responsive pathways intersect with immune, neuronal remodeling, and neurodegeneration-relevant mechanisms, including RNA methylation-associated immune signatures in MDD, multi-omics evidence implicating m6A in neuropsychiatric disorders, and c-Jun/AP-1 stress-signaling biology in neuronal injury and adaptation (Ren et al., 2024; Liufu et al., 2024; Khan et al., 2026). A study conducted in our lab revealed a link between stress and widespread changes in m6A methylation across the transcriptome. We examined the m6A methylation patterns in the prefrontal cortex (PFC) of rats subjected to chronic restraint stress (CRS). Our findings indicated that 4,301 transcripts were hypermethylated, while 79 were hypomethylated. Additionally, we conducted gene expression profiling, identifying 847 upregulated and 577 downregulated genes in CRS rats. By integrating m6A methylation data with our gene expression data, we identified 17 coding gene transcripts that were downregulated. Further functional analysis of these downregulated genes revealed their involvement in neuronal processes, including synaptic plasticity, chromatin remodeling, and neurotransmitter signaling. Although these studies are largely hypothesis-generating rather than mechanistic, they strengthen the view that m6A and related RNA methylation pathways may participate in MDD through immune regulation, transcriptomic remodeling, stress-response signaling, and disease-relevant gene networks.

Several sources of heterogeneity may help explain discrepant findings across studies, including differences in stress paradigm, species, brain region, sex, developmental timing, cell-type composition, assay platform, and statistical thresholds. Interpretation of YTHDF-family data is also evolving because recent models propose partial functional redundancy or cooperative action among YTHDF1, YTHDF2, and YTHDF3 rather than strictly isolated translation- or decay-specific roles (Zaccara and Jaffrey, 2020; Zaccara and Jaffrey, 2024). These unresolved issues emphasize the need for direct mapping of methylated transcripts, matched RNA and protein measurements, and perturbation experiments that test whether specific m6A readers mediate stress- or depression-relevant transcript fate.

RNA methylation-directed epitranscriptomic regulation remains an emerging field, and its molecular mechanisms in the brain are not yet fully defined. The involvement of the PFC and hippocampus in neural plasticity is well documented. However, in MDD pathophysiology, this plasticity-related feature of hippocampal synapses is strongly affected by disruptions in the regulation of related gene expression and in their availability within the synaptic environment. The precise regulatory mechanism behind this functional deficiency is poorly understood. Hence, it will be interesting to comprehend the role of this unique RNA-level modification in the complex gene regulatory system. Defining how RNA-level modifications regulate coding and non-coding transcripts within disease-relevant neural circuits may therefore provide important insight into the neuropathological basis of MDD (Figure 3).

## 4 MDD brain and m6A epitranscriptomics

Understanding the neurobiological complexity of major depressive disorder (MDD) remains a major priority because of its substantial global health burden. Recent estimates from the World Health Organization indicate that depressive disorders affect more than 300 million individuals worldwide, with a higher prevalence in women than in men (World Health Organization, 2017, 2025). The relatively limited therapeutic response to existing antidepressant treatments further underscores the need to identify mechanisms that can inform more effective strategies for this neuropsychiatric disorder (Rush et al., 2006). Earlier evidence from neuroimaging/neuropathological studies, along with preclinical models, has strongly indicated structural and functional abnormalities in corticolimbic brain areas in MDD subjects, including the prefrontal cortex, hippocampus, amygdala, and related limbic circuits (Dai et al., 2019; Pizzagalli and Roberts, 2022). These structural and functional deficits originating from related areas of the MDD brain have been associated with broad-spectrum changes in gene expression within the corticolimbic signaling network (Kang et al., 2007; Yoshino et al., 2021). Over the past decades, a plethora of reports on diverse genetic and epigenetic mechanisms have greatly advanced our understanding of their critical roles in the development of MDD and its associated pathophysiology (Saavedra et al., 2016; Penner-Goeke and Binder, 2019). It is interesting to note that the differential mode of gene regulation based on altered spatio-temporal context remains central to understanding this mental disorder, with far-reaching consequences for the cellular network at the neuronal level (Kang et al., 2007; Saavedra et al., 2016; Penner-Goeke and Binder, 2019; Yoshino et al., 2021). In this context, it has been shown that a disrupted cellular network underlies aberrant information processing in neuronal circuits that integrate numerous intra- and extracellular signaling axes (Dai et al., 2019; Yoshino et al., 2021). Data have also shown that this altered cellular network often adversely affects mood, cognition, and neurovegetative functions in the brain of individuals with MDD (Dai et al., 2019; Pizzagalli and Roberts, 2022). So far, most epigenetic studies related to the MDD brain have primarily focused on deciphering the roles of DNA methylation and histone-based modifications in gene expression regulation (Saavedra et al., 2016; Penner-Goeke and Binder, 2019). However, they have limited potential to directly influence the translational output by reversible transcript modification (Roundtree et al., 2017; Jiang et al., 2021). In this context, a detailed anatomical study that primarily examines potential dysregulation of the m6A RNA methylation machinery in relation to MDD pathology may further clarify the role of the intricate underlying neuro-molecular circuitry. Until recently, direct evidence for m6A dysregulation in the human MDD brain was limited. However, recent postmortem brain studies have now begun to demonstrate that m6A RNA methylation is altered in MDD-relevant cortical regions (Mitsuhashi et al., 2024; Roy and Dwivedi, 2025).

The first postmortem MDD brain study on m6A was conducted by Mitsuhashi et al. (2024). The study characterized m6A profiles in the ventromedial prefrontal cortex of individuals with MDD and healthy controls using m6A sequencing and identified more than 30,000 high-confidence m6A peaks that differed between MDD and control subjects. Among peaks that exceeded the significance threshold, m6A patterns exhibited opposing sex-specific profiles in MDD. In males with MDD, dysregulated m6A-modified transcripts were enriched for microtubule movement-related functions, whereas in females with MDD, altered m6A profiles were associated with neuronal projection-related processes.

Recently, data from our postmortem MDD subjects provided preliminary insight into the emerging role of this unique modification in regulating RNA dynamics in the cortical region (Roy and Dwivedi, 2025). We analyzed m6A methylation alterations in the dorsolateral prefrontal cortex of MDD subjects and non-psychiatric controls using a high-throughput m6A microarray approach. The analysis identified 1,290 significantly hypermethylated and 6,842 hypomethylated transcripts in MDD compared with controls. Most m6A sites were enriched within coding sequences, suggesting that m6A modification may affect transcripts involved in protein-coding gene regulation. Most interestingly, mRNA expression analysis revealed reduced FTO demethylase and increased METTL3 methyltransferase levels. However, because the study used bulk postmortem tissue and a microarray-based profiling platform, the results cannot resolve cell-type-specific methylation changes, distinguish neuronal from glial contributions, or by themselves establish causal relationships between m6A changes and MDD pathophysiology. The majority of m6A hypermethylated genes showed inverse correlation with their mRNA expression levels. As an additional analysis, we also compared m6A profiles between the MDD non-suicide and MDD suicide groups. We identified 6,750 transcripts with significant hypermethylation and 6,159 transcripts with significant hypomethylation in the MDD-suicide group. Of the hypermethylated genes, 196 were explicitly associated with suicide in the MDD patients, while 38 appeared to increase the risk of suicide in this group. These findings provide important but still hypothesis-generating evidence that altered m6A methylation may reshape the cortical epitranscriptome in MDD and require independent replication with orthogonal methods.

Collectively, the experimental evidence from these studies linking m6A modifications in the MDD brain provides a rationale for considering altered epitranscriptomic profiles as correlates of impaired cortical neuronal function (Mitsuhashi et al., 2024; Roy and Dwivedi, 2025). These studies are notable because they begin to directly profile m6A marks in human MDD brain tissue; however, the available human data remain limited by postmortem design, bulk-tissue averaging, potential confounding by medication, agonal state, postmortem interval, sex composition, comorbidity, and the current lack of extensive independent replication. This process may operate through altered reversible methylation and demethylation of many plasticity-related transcripts, leading to subsequent changes in their RNA stability, expression, and translational output in cortical regions of the MDD brain (Mitsuhashi et al., 2024; Roy and Dwivedi, 2025). Because some MDD-specific studies discussed in this review were conducted by members of the author group, we present those findings with attention to methodological limitations and the need for independent validation.

Cell-type specificity is a major unresolved issue. Bulk-tissue analyses may obscure divergent m6A signatures across excitatory pyramidal neurons, inhibitory interneurons, serotonergic neurons, corticotropin-releasing factor circuits, astrocytes, oligodendrocytes, oligodendrocyte precursor cells, microglia, endothelial cells, and infiltrating immune cells (Mitsuhashi et al., 2024; Roy and Dwivedi, 2025). Because these cell populations differ in RNA metabolism, stress responsivity, inflammatory signaling, synaptic support, myelination, and neurotrophin responsiveness, future work should combine single-nucleus or cell-sorted transcriptomics, spatial profiling, and direct RNA modification mapping to determine which cellular compartments drive MDD-associated m6A changes (Tegowski et al., 2024).

Methodological differences also influence interpretation. MeRIP/m6A-seq provides transcriptome-wide enrichment profiles but has limited resolution and depends on antibody specificity (Dominissini et al., 2012; Meyer et al., 2012; Zhang et al., 2022). miCLIP improves site-level resolution but requires specialized library preparation and can be affected by crosslinking efficiency (Zhang et al., 2022). DART-seq enables antibody-free detection through APOBEC-based editing but may introduce expression- or editing-dependent biases (Meyer, 2019; Zhu et al., 2022). m6A-SAC-seq provides high-resolution chemical-assisted mapping but requires careful optimization and sufficient RNA input. Direct RNA nanopore sequencing can detect native RNA modifications and isoforms but currently requires robust computational models and careful validation (Zhang et al., 2022). Microarray-based m6A profiling can screen many transcripts but is constrained by probe design, dynamic range, annotation coverage, and inability to discover unannotated sites. Therefore, convergent evidence across orthogonal platforms will be essential for validating disease-associated m6A signatures.

## 5 Translational potential of m6A epitranscriptomics in MDD: biomarkers and therapeutic opportunities

MDD is a complex disorder that still relies heavily on symptoms for diagnosis, with treatment largely based on empirical evidence. Despite advances in understanding the environmental, genetic, and molecular determinants of depression, reliable molecular biomarkers and mechanism-based therapeutic targets remain limited. Emerging evidence of epitranscriptomic regulation via m6A RNA methylation offers a promising avenue to address these limitations. M6A methylation is reversible and mediates the interface between environmental influences and the regulation of gene expression, providing an opportunity to develop biomarkers and therapeutic interventions. Recent studies of various neurological disorders have revealed altered expression of m6A-related enzymes, including METTL3, METTL14, FTO, and ALKBH5, in blood cells, serum, and extracellular vesicles (Ge et al., 2023; Lei and Wang, 2022; Mao et al., 2023; Liufu et al., 2024). These findings suggest that similar approaches may be applicable in MDD. Postmortem MDD brain studies have shown a significant reduction in FTO expression, while METTL3 and METTL14 expression were upregulated. This leads to alterations in m6A modifications of transcripts involved in axon guidance and neuroplasticity pathways. It is suggested that this epitranscriptomic signature, which is present in the brain, may also be present in peripheral tissues, including peripheral blood mononuclear cells (PBMCs) and serum-derived extracellular vesicles.

Beyond its diagnostic utility, m6A methylation presents a biologically attractive but still early therapeutic target. Its reversibility enables longitudinal assessment of m6A patterns before and after antidepressant treatment, potentially providing molecular indicators of therapeutic response. Because m6A methylation is regulated by writers, erasers, and readers, dysregulation of FTO, METTL3, and METTL14 may perturb axon guidance, synaptic plasticity, neuronal signaling, and stress-response pathways implicated in depression. Restoring balanced m6A methylation may therefore represent a novel therapeutic strategy. Potential approaches include modulating erasers such as FTO and ALKBH5 to normalize transcript turnover and translation of synaptic plasticity-associated genes; however, most available small-molecule inhibitors and degraders have been developed in oncology contexts rather than psychiatric disease (Huang et al., 2019; Su et al., 2020; Li et al., 2025). Before therapeutic translation to MDD, key barriers include CNS penetrance, pharmacokinetics, toxicity, transcriptome-wide specificity, target engagement in relevant brain regions and cell types, and the risk of disrupting essential RNA regulatory programs. A complementary strategy involves inhibiting or enhancing METTL3 and METTL14, which regulate stress-responsive transcripts and synaptic networks, although achieving transcriptome-wide specificity remains challenging. Targeting m6A reader proteins may offer greater functional selectivity, as these proteins determine the fate of methylated mRNAs; nevertheless, no psychiatric clinical data yet establish the safety or efficacy of directly targeting m6A writers, erasers, or readers. For example, inhibition of YTHDF2 could theoretically reduce degradation of transcripts relevant to depression-related molecular pathways, but this remains a preclinical hypothesis requiring rigorous validation.

Non-coding RNAs represent another important frontier. m6A modification can regulate long non-coding RNAs, circular RNAs, pri-miRNAs, and miRNA biogenesis, thereby influencing chromatin regulation, RNA sponging networks, synaptic gene expression, inflammatory signaling, and stress-responsive transcriptional programs (Alarcón et al., 2015; Liu and Xiang, 2023; Lee et al., 2025). These mechanisms are relevant to depression because lncRNAs, circRNAs, and miRNAs have been implicated in neuroplasticity, HPA-axis regulation, immune activation, and antidepressant response. However, direct evidence linking m6A-modified non-coding RNAs to human MDD remains limited, and future studies should map m6A changes on coding and non-coding RNA species in parallel.

## 6 Conclusions and future perspective

Future studies should move beyond global measurements of RNA methylation and systematically define transcript-, cell-type-, region-, and sex-specific m6A landscapes in well-characterized cohorts of individuals with MDD. Such studies should focus on corticolimbic regions implicated in depression, including the dorsolateral prefrontal cortex, ventromedial prefrontal cortex, hippocampus, anterior cingulate cortex, amygdala, and nucleus accumbens. For example, recent postmortem human studies have shown altered m6A methylation in the ventromedial prefrontal cortex, with sex-dependent differences in pathways related to microtubule motility in males and neuronal projections in females. Similarly, our postmortem dorsolateral prefrontal cortex data indicate widespread m6A remodeling in MDD, including reduced FTO expression, increased METTL3 expression, and altered methylation of transcripts involved in neuroplasticity, axon guidance, and suicide-risk-associated pathways. These findings highlight the need to determine whether m6A dysregulation represents a primary pathogenic mechanism, a compensatory adaptation to chronic stress, or a downstream consequence of altered neuronal activity.

Mechanistic studies will be essential to establish causal links between m6A dysregulation and depression-related phenotypes. Specific examples include examining whether stress-induced downregulation of FTO or upregulation of METTL3 alters the stability, localization, or translation of plasticity-related transcripts such as BDNF, NTRK2, CREB1, NR3C1, GRIN1, GRIN2A, GRIN2B, CAMK2A, SYN1, and ARC. In learned helplessness and chronic stress models, altered m6A marking of glucocorticoid signaling and neurotrophin-related transcripts may influence synaptic remodeling in the hippocampus and prefrontal cortex. Similarly, FTO-dependent regulation of ADRB2 mRNA in the hippocampus provides a concrete example of how m6A demethylation can influence depression-like behavior through a defined transcript. Future work should therefore combine m6A-seq, miCLIP, nanopore direct RNA sequencing, RNA-seq, ribosome profiling, and proteomics to determine whether altered m6A marks change transcript abundance, translational efficiency, or protein output.

From a translational perspective, m6A-based signatures may serve as useful biomarkers for diagnosis, stratification, treatment response, and suicide risk prediction. Candidate peripheral sources include peripheral blood mononuclear cells, serum or plasma extracellular vesicles, platelet RNA, and circulating cell-free RNA. For example, peripheral assessment of FTO, METTL3, METTL14, WTAP, ALKBH5, YTHDF1, YTHDF2, YTHDC1, YTHDC2, and IGF2BP family members could be paired with clinical measures of symptom severity, treatment resistance, inflammatory burden, HPA-axis dysregulation, or suicidal behavior. Longitudinal studies before and after selective serotonin reuptake inhibitors, serotonin-norepinephrine reuptake inhibitors, ketamine, electroconvulsive therapy, repetitive transcranial magnetic stimulation, psychotherapy, or anti-inflammatory interventions could determine whether m6A signatures normalize with clinical improvement or predict relapse. A particularly informative design would compare baseline and post-treatment m6A profiles in responders and non-responders, while simultaneously measuring candidate transcripts, including BDNF, NTRK2, FKBP5, NR3C1, SLC6A4, TPH2, IL6, TNF, and CRP-related inflammatory networks.

The reversible nature of m6A modification also makes the pathway attractive for therapeutic exploration. Potential strategies include modulating eraser activity, such as FTO or ALKBH5, to restore abnormal transcript turnover; targeting writer-complex components such as METTL3, METTL14, WTAP, VIRMA, ZC3H13, HAKAI, or RBM15 to rebalance methylation at stress-responsive transcripts; or regulating reader proteins such as YTHDF1, YTHDF2, YTHDF3, YTHDC1, YTHDC2, and IGF2BPs to alter the downstream fate of methylated RNAs. For example, reducing excessive YTHDF2-mediated decay could theoretically preserve transcripts important for synaptic resilience, whereas enhancing YTHDF1-dependent translation might restore protein synthesis required for adaptive plasticity. However, because m6A regulators influence thousands of transcripts and available inhibitors are largely oncology-focused, therapeutic development for MDD must prioritize brain-region specificity, cell-type specificity, CNS exposure, dose precision, toxicity monitoring, target engagement, and avoidance of broad transcriptome disruption. At present, these strategies should be viewed as long-term translational possibilities rather than clinically validated psychiatric interventions.

Patient-derived experimental systems will be especially valuable for testing these possibilities. MDD patient-derived skin fibroblasts can be used as an accessible cellular platform to examine baseline differences in m6A regulators, stress-hormone responsiveness, inflammatory stimulation, mitochondrial function, and antidepressant-induced molecular changes. These fibroblasts could also be reprogrammed into induced pluripotent stem cells and differentiated into excitatory neurons, inhibitory interneurons, astrocytes, microglia-like cells, or three-dimensional brain organoids. Such models would allow direct testing of whether manipulating FTO, METTL3, METTL14, or YTHDF2 rescues stress-induced changes in synapse formation, dendritic spine density, neurotrophin signaling, glucocorticoid receptor responsiveness, or inflammatory gene expression. Combining these models with CRISPR-based perturbation, single-cell RNA sequencing, spatial transcriptomics, and direct RNA modification mapping could identify cell-type-resolved mechanisms through which m6A dysregulation contributes to MDD vulnerability.

In conclusion, m6A RNA methylation provides a biologically plausible and experimentally tractable framework for linking environmental stress, altered gene regulation, impaired synaptic plasticity, and neural circuit dysfunction in MDD. Specific examples from human postmortem cortex, learned helplessness models, chronic stress paradigms, and genetic association studies collectively suggest that m6A dysregulation may affect pathways involving FTO, METTL3, METTL14, ALKBH5, YTH-domain readers, glucocorticoid signaling, serotonergic regulation, neurotrophin signaling, immune activation, and synaptic remodeling. Although the field remains in its early stages, emerging evidence supports the view that m6A-based epitranscriptomic regulation may be both a mechanistic contributor to MDD pathophysiology and a potential source of biomarkers and therapeutic targets. Future progress will require integrated human, animal, and patient-derived cellular studies designed to establish causality, validate clinical utility, and determine whether targeted manipulation of this reversible RNA regulatory system can restore adaptive gene expression in the depressed brain.
